# Autoimmunity and Otolaryngology Diseases

**DOI:** 10.1155/2018/2747904

**Published:** 2018-10-04

**Authors:** Massimo Ralli, Arianna Di Stadio, Armando De Virgilio, Adelchi Croce, Marco de Vincentiis

**Affiliations:** ^1^Department of Sense Organs, Sapienza University of Rome, Rome, Italy; ^2^San Camillo Hospital IRCCS, Venice, Italy; ^3^Humanitas Clinical and Research Center, Via Alessandro Manzoni, 56-20089 Rozzano, Italy; ^4^Department of Otolaryngology, Università Gabriele d'Annunzio Chieti, Italy; ^5^Department Oral and Maxillofacial Sciences, Sapienza University of Rome, Rome, Italy

## 1. Main Text

Many systemic autoimmune diseases have otolaryngology manifestations that could represent a diagnostic challenge for clinicians, as they often constitute an early sign of an otherwise asymptomatic autoimmune condition and may lead to delayed diagnosis and treatment. In other cases, otolaryngology manifestations can be overlooked in patients with previously diagnosed autoimmune diseases. The presence of concomitant conditions, the heterogeneity of studies available in the literature, and the lack of randomized trials are factors that may limit the prompt diagnosis of otolaryngology manifestations in systemic autoimmune diseases, with underestimation of the problem and undertreatment of the related condition.

Audio-vestibular symptoms may be found in a variety of autoimmune diseases, and early diagnosis is essential for the elevate chances of near-complete restoration when specific therapy is promptly initiated. Sensorineural hearing loss is the most common audiological symptom associated to systemic autoimmune diseases, although conductive hearing loss may also be present. Hearing loss may present in a sudden, slowly or rapidly progressive, or fluctuating form and is mainly bilateral and asymmetric. Current evidence shows a good response of hearing impairment to corticosteroid therapy that may lead to near-complete hearing restoration. Vestibular symptoms, tinnitus, and aural fullness often mimic Menière's disease in patients with systemic autoimmune conditions and mainly affect both ears simultaneously. Inner ear involvement in autoimmune diseases is suggested by the history, clinical findings, an immunologic evaluation of the patient's serum, and response to immunosuppressive therapies, following exclusion of other known causes. Hearing loss, vertigo, and tinnitus, as reported in the reviews by Ralli et al. and Girasoli et al., can be found in patients with Wegener's granulomatosis, systemic lupus erythematosus, Cogan's syndrome, relapsing polychondritis, polyarteritis nodosa, Sjögren's syndrome, myasthenia gravis, Behçet's disease, Takayasu's arteritis, rheumatoid arthritis, and other autoimmune conditions.

Oral manifestations, such as recurrent oral mucosal ulcerations, can be seen in patients with systemic lupus erythematosus, Sjögren's syndrome, pemphigus vulgaris, mucous membrane pemphigoid, and Behçet's disease, as described in the paper by Saccucci et al. In these cases, the dentist plays a central role to reach an early diagnosis and therefore improving the quality of treatment strategies as well as the quality of life in affected patients.

Salivary gland involvement may be found in patients with Sjögren's syndrome and sarcoidosis, two conditions that may also be associated to xerostomia, trigeminal nerve dysfunction, and peripheral facial nerve palsy. Furthermore, salivary glands can be affected in the rare IgG4-related disease; the article from Puxeddu et al. discusses new insights in the pathogenesis of IgG4-related disease with involvement of the salivary glands, focusing on its clinical aspects and the tools that are currently available for a correct differential diagnosis with other conditions affecting the salivary glands.

Nose and paranasal sinuses can be affected in patients with Wegener's granulomatosis, Churg-Strauss syndrome, polychondritis, and sarcoidosis. Laryngeal involvement with cricoarytenoid joint alterations can be found in rheumatoid arthritis, ankylosing spondylitis, and gout. Dysphagia has been described in patients with dermatomyositis, systemic sclerosis, and systemic lupus erythematosus.

The aim of this special issue was to stimulate publication of research, both in the form of original articles and review papers, to describe the current state of the art of otolaryngology manifestations in systemic autoimmune diseases. Many manuscripts that focused on different topics within the field of this special issue were submitted, and after a thorough peer review process, seven papers were accepted for publication.

We hope that this special issue will provide valuable information to interested researchers and clinicians and will raise awareness on otolaryngology manifestations in autoimmune systemic disease to favor an early diagnosis and appropriate treatment of these conditions.

